# Early impact of maternal RSV vaccination on bronchiolitis hospitalisation and critical care admissions in infants under age 3 months, England, September 2024 to March 2026

**DOI:** 10.2807/1560-7917.ES.2026.31.36.2600752

**Published:** 2026-09-10

**Authors:** Jonathan Broad, Matthew Wilson, Jaime Borjas-Howard, Merryn Voysey, Simon B Drysdale, Nick Andrews, Conall H Watson, Heather J Whitaker

**Affiliations:** 1 Department of Immunisation and Vaccine-Preventable Diseases, UK Health Security Agency, London, United Kingdom; 2 Oxford Vaccine Group, Department of Paediatrics, University of Oxford, Oxford, United Kingdom; 3 The NIHR Oxford Biomedical Research Centre, Oxford, United Kingdom; 4 Health Protection Research Unit in Respiratory Infections, Imperial College, London, United Kingdom

**Keywords:** RSV, bronchiolitis, vaccine, maternal, hospitalisation, infants

## Abstract

We conducted a time series analysis of hospitalisation and critical care admissions to evaluate the impact of maternal respiratory syncytial virus (RSV) vaccination in England from September 2024 to March 2026 in infants < 3 months of age. All bronchiolitis admissions decreased by 39.3% (IRR: 0.607, 95% CI: 0.576–0.640), and RSV bronchiolitis admissions by 62.6% (IRR: 0.374, 95% CI: 0.344–0.407). We estimated a 41.0% reduction in bronchiolitis critical care admissions (IRR: 0.590, 95% CI: 0.531–0.657). Maternal vaccine coverage was 55.1%. These findings support efforts to expand vaccine uptake.

Real-world data evaluating the impact of maternal RSV vaccine programmes on bronchiolitis hospitalisations in young infants are limited, particularly among those born preterm [1]. In September 2024, the United Kingdom (UK) implemented a year-round universal maternal RSV vaccine offered from 28 weeks’ gestation [2]. Maternal RSV vaccine coverage for infants born in England had reached 64.1% by November 2025; although across the whole study period, 55.1% of births were infants born to vaccinated mothers [3]. Here, we aimed to evaluate population-level changes in bronchiolitis hospital and critical care admissions in England in term and preterm infants under 3 months of age between 1 January 2015 and 31 March 2026, following maternal RSV vaccination using a retrospective interrupted time series analysis. 

## Approach to interrupted time series

The entire study population comprised infants aged 0 to 365 days admitted to the hospital between 1 January 2015 and 31 March 2026 with (i) all bronchiolitis (ICD-10 [4] diagnostic code J21.0–J21.9), (ii) RSV-specific bronchiolitis (J21.0), or (iii) non-confirmed bronchiolitis (J21.1, J21.8, J21.9). Non-confirmed bronchiolitis referred to bronchiolitis without a specific RSV diagnostic code, in which RSV was not found or not tested for, rather than a disease confirmed to have a non-RSV aetiology. It includes human metapneumovirus (J21.1) which may or may not be amenable to RSV vaccination, and other specified organisms (J21.8), although most events are not pathogen specific (J21.9) (15). ICD-10 codes were assigned at the end of a completed hospital episode. Bronchiolitis was identified only if recorded by the treating clinician and captured in the primary or top two secondary codes in the Hospital Episode Statistics (HES) database. 

The maternal RSV vaccine eligibility was based on date of birth: infants born before 1 September 2024 were classified as pre-vaccination; infants born between 1 September and 26 October 2024 during the first transitional 8 weeks of the vaccination programme were classified separately and excluded from the main analysis; infants born 27 October 2024 onwards were classified as post-vaccination. Prematurity (< 37 weeks’ gestational age) was ascertained from Maternity Services DataSet (MSDS) where available, otherwise on ICD-10 codes. Critical care admissions were identified from the HES critical care table. Immunisation with nirsevimab was provided to high-risk children as per the UKHSA Green Book recommendations and these children were included in the study irrespective of maternal vaccination status [5]. To assess the potential contribution of nirsevimab immunisation to prevent infant hospitalisation for bronchiolitis, a sensitivity analysis was conducted only evaluating term infants who were not eligible for nirsevimab.

Individual data were aggregated to give weekly counts of hospital, critical care and preterm admissions, by mothers’ RSV vaccine eligibility status and infant age group (0–2, 3–5 and 6–11 months). Weekly counts were analysed in R (version 4.5.3) with a quasi-Poisson model, with explanatory variables for mothers’ RSV vaccine eligibility x age group interaction (variable of interest among ages 0–2 months), calendar week (factor) x age group interaction (to allow for regular seasonal trends), year (continuous 1,2,...,12, to account for long-term trends), and RSV season year (continuous, 0 outside season, 1,2,...,12 in season weeks 46–3) (to allow long-term trends during RSV season to differ from those outside season), plus offset. We removed COVID-19 pandemic months from the model due to disrupted epidemiology (16 Mar 2020–30 Sep 2022). Inclusion of older infants aged 3–5 months, and 6–11 months aided estimation of long-term trends. Infants aged 3–5 months were included in the analysis of hospitalisations averted; further evaluation of age-specific trends will be presented in future analyses. Offsets were based on live birth registration statistics from the Office of National Statistics and the proportion of infants classified to each vaccine eligibility group, age group and week, similar to methods described elsewhere [6,7]. The pre-vaccination period was the reference category for the impact estimates outlined here; the 8-week transition was modelled separately (data available on request) and not used to compare because vaccine uptake increased rapidly during this period and the effect of maternal vaccination in utero on infant outcomes was not immediate. A sensitivity analysis included an additional quadratic year term. This produced consistent results.

## Hospital admissions before maternal RSV vaccination introduction

Between 1 January 2015 and 31 August 2024, there were 137,803 bronchiolitis admissions among infants under 3 months of age in England with bronchiolitis where relevant ICD code data were available, of which 21,278 (15.4%) were preterm (< 37 weeks gestation). The seasonal average number of cases in the pre-vaccination period was 15,208 admissions. Between 1 January 2017 (when critical care records began) and 31 August 2024, there were 6,355 critical care bronchiolitis admissions among infants under 3 months of age.

## Hospital admissions after maternal RSV vaccination introduction

Following the introduction of maternal RSV vaccination up until 31 March 2026, hospital admissions for bronchiolitis in infants under 3 months of age decreased by 39.3% (IRR: 0.607, 95% CI: 0.576–0.640). Reductions were seen for RSV bronchiolitis (62.6% reduction; IRR: 0.374, 95% CI: 0.344–0.407) and non-confirmed bronchiolitis (31.4% reduction; IRR: 0.686, 95% CI: 0.648–0.727) (Figure;Table). This is equivalent to an estimated 9,856 bronchiolitis hospital admissions averted in infants under 3 months of age. As our model also included infants aged 3–5 months, this allowed us to estimate hospital admissions averted in infants aged under 6 months to provide a measure of broader potential impact. Applying the model to infants aged under 6 months, an estimated 16,255 hospital admissions were averted. 

**Figure:**
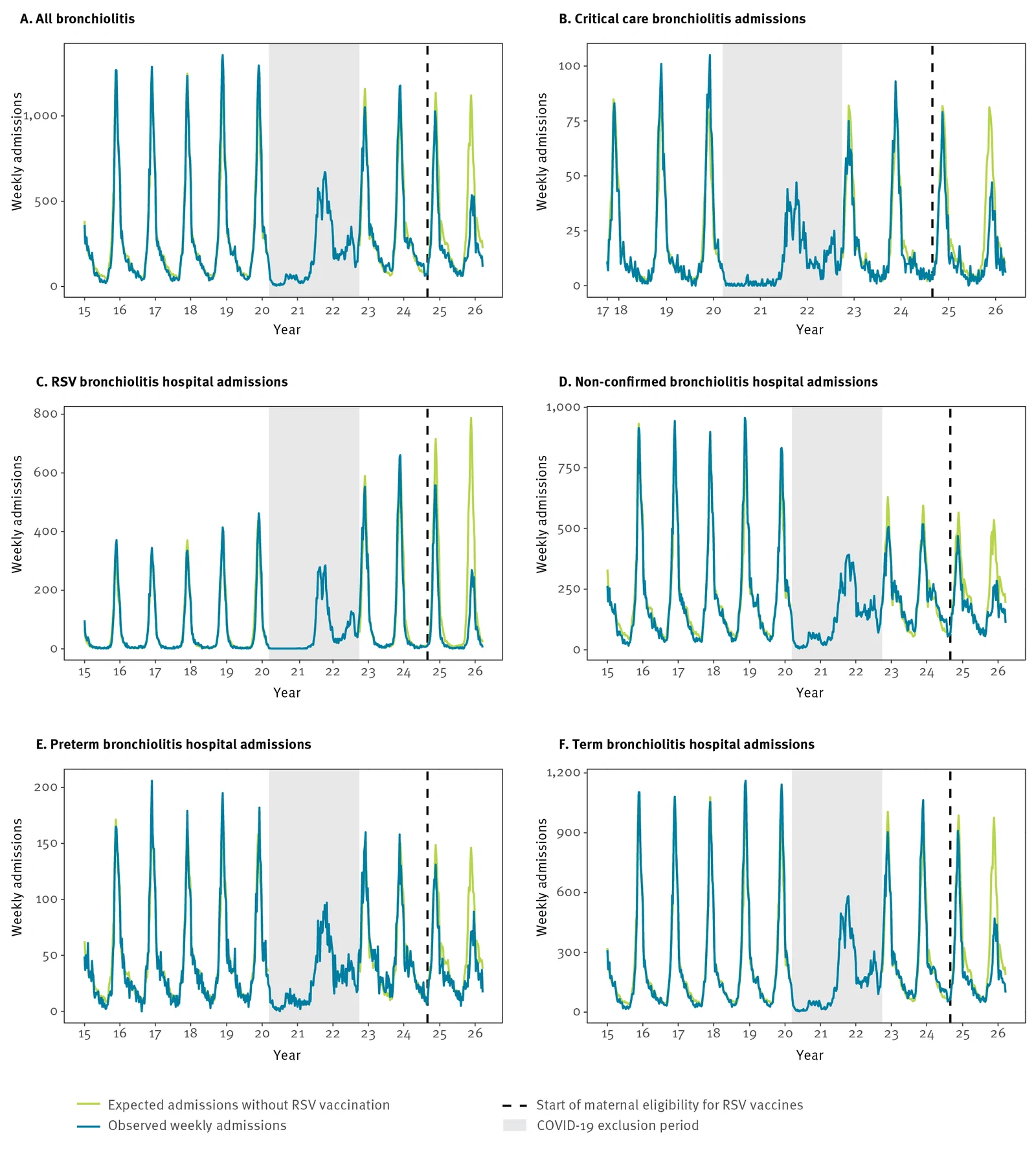
Observed bronchiolitis hospital and critical care admissions and predicted admissions in the absence of maternal RSV vaccination in infants under 3 months of age before and after the introduction of the national RSV vaccination campaign, England, 2015–2026

RSV: respiratory syncytial virus.

Bronchiolitis hospital and critical care admissions in infants aged under 3 months before and after the introduction of the national maternal RSV vaccination campaign in England using the primary quasi-Poisson seasonally adjusted regression. Infants born 1 January 2015 to 31 August 2024 were classified as pre-vaccination; infants born during the transitional 8 weeks of the programme (1 Sep– 26 Oct 2024) were modelled separately; infants born 27 October 2024 to 31 March 2026 were classified as post-vaccination. ‘All bronchiolitis’ refers to all episodes with a clinical diagnosis of bronchiolitis, irrespective of aetiology. ‘RSV bronchiolitis’ refers to episodes with an RSV-specific code, assumed to reflect laboratory confirmation in routine National Health Service testing. ‘Non-confirmed bronchiolitis’ refers to bronchiolitis without a specific RSV diagnostic code, in which RSV was not detected, or RSV testing was not conducted. Preterm refers to infants born before 37 weeks gestation; term refers to infants born at 37 weeks gestation or later. Panel F represents a sensitivity analysis restricted to term infants who were generally not eligible for nirsevimab prophylaxis in the UK.

The blue^†^ line represents observed weekly admissions. The light green^†^ line shows the predicted trend based on pre-vaccine data, demonstrating expected admissions in the absence of maternal RSV vaccination. The black dashed line represents the start of maternal eligibility for RSV vaccines (1 Sep 2024). The grey shaded areas represent the COVID-19 exclusion period (16 Mar 2020–30 Sep 2022).

**Table:** Model estimates and goodness-of-fit statistics for bronchiolitis admission outcomes reductions following maternal RSV vaccination introduction, with incidence rate ratios presented for infants aged under 3 months, England, 2015–2026

Outcome	Model	IRR	95% CI	p value	RMSE	MAE
All bronchiolitis hospital admissions	Linear seasonal	0.607	0.576–0.640	< 0.001	49.40	31.96
All bronchiolitis hospital admissions	Quadratic seasonal	0.655	0.619–0.694	< 0.001	48.78	31.73
Critical care bronchiolitis admissions	Linear seasonal	0.590	0.531–0.657	< 0.001	4.88	3.15
Critical care bronchiolitis admissions	Quadratic seasonal	0.620	0.545–0.705	< 0.001	4.86	3.14
Preterm bronchiolitis hospital admissions	Linear seasonal	0.642	0.600–0.687	< 0.001	9.37	6.59
Preterm bronchiolitis hospital admissions	Quadratic seasonal	0.706	0.656–0.760	< 0.001	9.25	6.51
RSV bronchiolitis hospital admissions	Linear seasonal	0.374	0.344–0.407	< 0.001	23.99	12.21
RSV bronchiolitis hospital admissions	Quadratic seasonal	0.357	0.324–0.393	< 0.001	23.90	12.15
Non-RSV-confirmed bronchiolitis hospital admissions	Linear seasonal	0.686	0.648–0.727	< 0.001	39.46	26.15
Non-RSV-confirmed bronchiolitis hospital admissions	Quadratic seasonal	0.773	0.727–0.822	< 0.001	37.55	25.37

CI: confidence interval; IRR: incidence rate ratio; MAE: mean absolute error; RMSE: root mean square error.

IRRs are presented for bronchiolitis hospital and critical care admissions in infants aged under 3 months using a primary quasi-Poisson seasonally adjusted regression and secondary quasi-Poisson quadratic-adjusted regression. Infants born before 1 September 2024 were classified as pre-vaccination, those born during the transitional 8 weeks of the programme (1 Sep–26 Oct 2024) were modelled separately; infants born from 27 October 2024 were classified as post-vaccination. The IRRs compare the post-vaccination period with the pre vaccination period. Lower RMSE and MAE suggest better model fit to the observed data. P values below 0.05 are considered statistically significant. Full model descriptions are available in Supplementary Table S3. The model also included infants aged 3–5 months, thereby allowing us to estimate hospital admissions averted 3–5 months. We additionally report the estimated number of admissions averted in infants aged under 6 months to provide a measure of broader potential impact.

We conducted a separate sensitivity analysis using a quadratic model to assess potential non-linear temporal trends, the full model outputs and full admissions averted are provided in Supplementary Figure S1, Table S1, and Table S2 respectively. Model development is outlined in Supplementary Table S3. Based on descriptive analysis of the weekly case data, the bronchiolitis season in 2025/26 was both reduced and later than years 2015–2024, consistent with the pattern shown in the Figure; 50% of seasonal cases occurred by week 51 compared with week 48–49 in previous years. Precise median weeks data are not shown. Critical care bronchiolitis admissions were reduced by 41.0% (IRR: 0.590, 95% CI: 0.531–0.657). Among preterm infants (< 37 weeks), bronchiolitis hospital admissions declined by 35.8% (IRR: 0.642, 95% CI: 0.600–0.687). 

## Discussion

Respiratory syncytial virus (RSV) contributes to 1.4 million hospital admissions in children under 6 months of age every year globally, with over 500,000 admissions among preterm infants [8,9]. Respiratory syncytial virus immunisation programme evaluations to date have focussed on immunisation of children with nirsevimab, a long-acting monoclonal antibody [10-12]. This contrasts with maternal RSV vaccination, which aims to protect young infants via transplacental transfer of maternal antibodies. The UK introduced maternal RSV vaccination in England in September 2024 with a protein-based vaccine, ABRYSVO (Pfizer). Over 520,000 doses of maternal RSV vaccine were administered in England during the study period; however, some pregnancies had not yet resulted in a birth by the end of follow-up [13]. Palivizumab was replaced by nirsevimab in September 2025 for high-risk infants, with eligibility expanded to include infants born before 32 weeks’ gestation irrespective of maternal vaccination status; approximately 9,000 doses were used across the UK in the 2025/26 season [5]. 

Introduction of maternal RSV vaccination was associated with significant reductions in hospital and critical care admissions. We observed a 39.3% reduction in hospital admissions for all bronchiolitis among infants under 3 months of age in two seasons, when maternal vaccine coverage reached a peak of 64% [3]. Similar effects were observed for critical care admissions (41.0%), and bronchiolitis hospital admissions among preterm infants (35.8%). When restricting to RSV bronchiolitis, the impact was 62.6%. Bronchiolitis that was not coded as RSV fell by 31.4%. The reduction in non-RSV confirmed bronchiolitis may reflect reductions in bronchiolitis associated with other pathogens or changes in diagnostic attribution.

These data are consistent with real-world data from Argentina and the United States (US). In Buenos Aires, Argentina, as at November 2024, maternal RSV vaccination coverage was 55.3% and vaccine effectiveness was 66.1%; hospitalisations for bronchiolitis were reduced by 33.6% in infants under 6 months [1,14,15]. In the US during the 2024/25 season, a hybrid maternal and nirsevimab programme achieved an estimated combined uptake of 60–65% during peak circulation [1]. Maternal vaccine effectiveness against RSV bronchiolitis hospitalisation was 70%; RSV-associated hospitalisations were reduced by 41–51% in infants aged under 12 months and reduced by 56–63% in infants aged under 3 months [1,14,15]. Our results extend these findings by providing population-level evidence in a European setting using the World Health Organization’s vaccine schedule [16]. Findings are consistent with UK Health Security Agency (UKHSA) surveillance showing reduced bronchiolitis emergency department attendances and bronchiolitis admissions in 2025/26 compared with previous seasons, although ascertainment and methods differ [17]. The magnitude of RSV bronchiolitis reduction was substantial relative to the vaccine uptake, which may be affected by the addition of nirsevimab, prevention of repeat hospital admissions, or changes in testing; uptake may not be independent of baseline risk. The analyses are affected by vaccination uptake and are not a measure of vaccine effectiveness. This provides useful data for programme implementation.

These findings on RSV hospitalisation in young infants after the introduction of the maternal RSV vaccination programme are encouraging, and are strengthened by the use of national-level data, a long period before vaccine introduction, and the ability to study preterm infants [9]. Reductions in RSV bronchiolitis admissions could be explained by other changes such as testing behaviour changes. Changes in respiratory virus testing following the COVID-19 pandemic may have influenced the trends observed over time for RSV-confirmed and non-confirmed bronchiolitis hospital admissions; models (not shown) allowing separate pre- and post-pandemic trends produced broadly similar estimates. Some temporal trends in the data pre-dated the pandemic; we have therefore retained the longer-term trend in the primary analysis. Changes in testing may have influenced the distribution of RSV-confirmed cases over time. Individual-level immunisation data were not included. Palivizumab was switched to nirsevimab as prophylaxis for high-risk infants under 32 weeks (approximately 1.2% of births) during the study period, which could reduce hospital admissions, although on a limited scale compared with maternal vaccination [6]. We cannot completely separate the contribution of maternal vaccination and nirsevimab among eligible infants. While 9,000 doses of nirsevimab were given to infants during this study, over 520,000 doses of maternal vaccine were given by 31 March 2026, and when restricting to term infants who were not eligible for nirsevimab, the effects were similar. This provides reassurance that the observed population-level reduction was not restricted to groups eligible for infant monoclonal antibody prophylaxis and supports an impact of the maternal vaccination programme. Furthermore, we were unable to distinguish bronchiolitis associated with other pathogens in this analysis, such as human metapneumovirus. Non-RSV pathogen specific analyses were beyond the scope of the extracted dataset and the early evaluation, and this will be explored in future analyses. We plan further analyses spanning additional seasons and older infants.

## Conclusion

The introduction of universal maternal RSV vaccination in England was associated with a 39.3% reduction in bronchiolitis hospital admissions and 41.0% reduction in critical care admissions in infants under 3 months of age. These findings support efforts to expand vaccine uptake, optimise equitable access, and monitor effectiveness and impact among term and preterm infants including potential indirect protective effects of vaccination. Evaluation is needed to understand the effect of timing of vaccination, introduction of nirsevimab, changes to vaccine programme delivery, and whether effects are sustained over time.

## Data Availability

The underlying data used in this analysis are held by the UK Health Security Agency (UKHSA). We do not have the necessary permissions to share the data publicly or with third parties.

## †Erratum

At publication of this article, the line colours for the figure were mislabelled in the note. This was corrected on 14 September 2026. We apologise for any inconvenience this may have caused.

## Supplementary Data

488000 Supplement
