# Erratum for Euro Surveill. 2026;31(36)

**DOI:** 10.2807/1560-7917.ES.2026.31.37.260917e

**Published:** 2026-09-17

**Authors:** Eurosurveillance editorial team

**Affiliations:** 1 European Centre for Disease Prevention and Control (ECDC), Stockholm, Sweden

In the article ‘Early impact of maternal RSV vaccination on bronchiolitis hospitalisation and critical care admissions in infants under age 3 months, England, September 2024 to March 2026’ by Broad et al., published on 10 September 2026, the line colours for the figure were mislabelled in the note. This was corrected on 14 September 2026. We apologise for any inconvenience this may have caused.

