# Supplementary material for: Early impact of maternal RSV vaccination on bronchiolitis hospitalisation and critical care admissions in infants under age 3 months, England, September 2024 to March 2026
Source: Euro Surveill. 2026 Sep 10;31(36):2600752. doi: 10.2807/1560-7917.ES.2026.31.36.2600752 (PMC13570235; doi:10.2807/1560-7917.ES.2026.31.36.2600752)
Supplement: Supplementary file 1 [file 26-00752_BROAD_Supplement.pdf]

Supplementary material for: Early impact of maternal RSV vaccination on bronchiolitis hospitalisation and critical care admissions in newborn infants up to 3 months of age, England 2024-2026

Disclaimer: This supplementary material is hosted by Eurosurveillance as supporting information alongside the article 'Early impact of maternal RSV vaccination on bronchiolitis hospitalisation and critical care admissions in newborn infants up to 3 months of age, England 2024-2026' on behalf of the authors who remain responsible for the accuracy and appropriateness of the content. The same standards for ethics, copyright, attributions and permissions as for the article apply. Eurosurveillance is not responsible for the maintenance of any links or email addresses provided therein

Figure S1 Sensitivity analysis: Observed bronchiolitis hospital and critical care admissions (orange line) and predicted admissions in the absence of RSV vaccination (blue line) in infants under 3 months of age before and after the introduction of the national RSV vaccination campaign in England, estimated using a secondary quasi-Poisson seasonally and quadratic-adjusted regression analysis

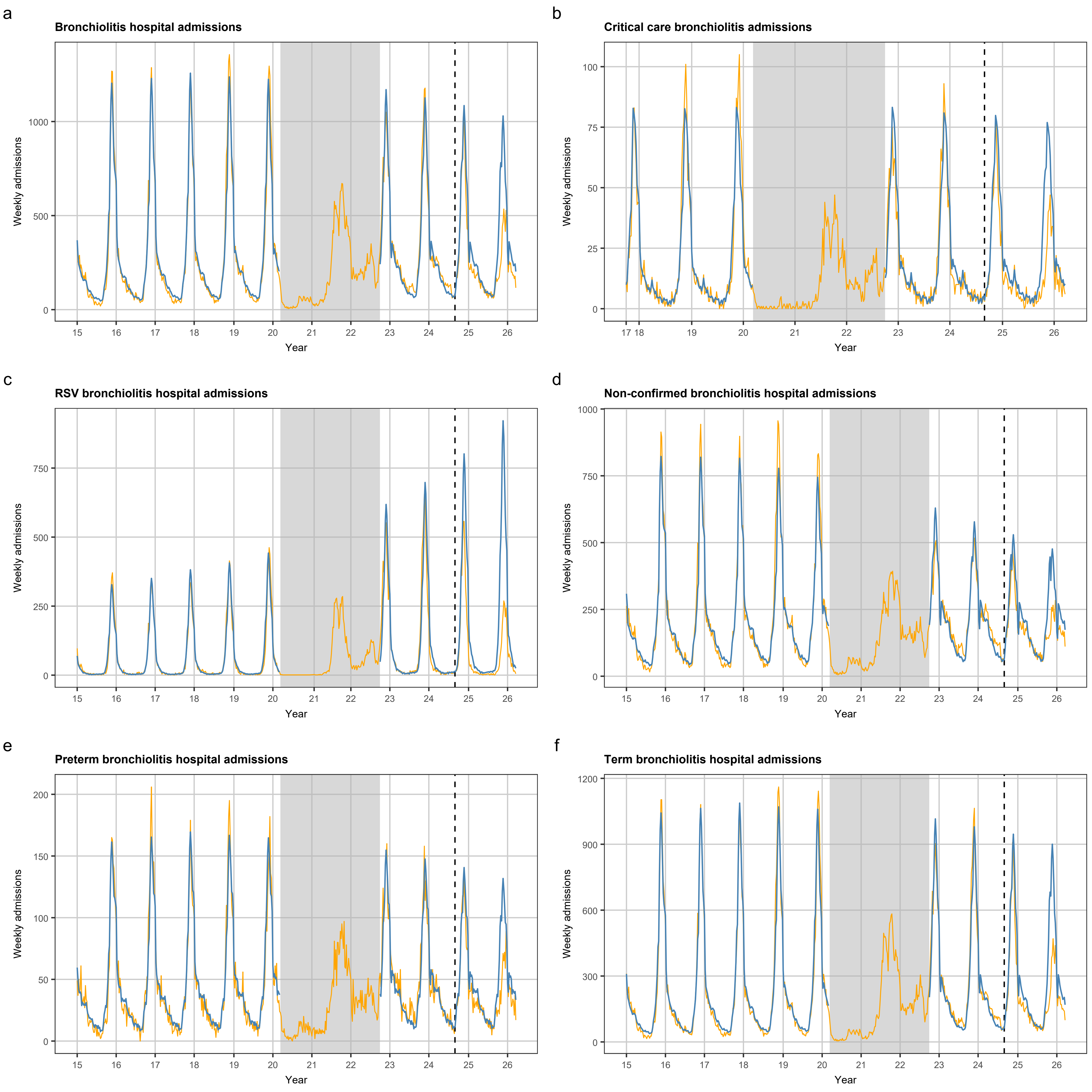
a
Bronchiolitis hospital admissions
b
Critical care bronchiolitis admissions
c
RSV bronchiolitis hospital admissions
d
Non-confirmed bronchiolitis hospital admissions
e
Preterm bronchiolitis hospital admissions
f
Term bronchiolitis hospital admissions
Weekly admissions
Year

Legend: Sensitivity analysis. Bronchiolitis hospital and critical care admissions in infants under 3 months before and after the introduction of the national RSV vaccination campaign in England using the secondary quasi-Poisson seasonally and quadratic-adjusted regression. Figure 1A) shows all bronchiolitis hospital admissions; 1B) shows critical care bronchiolitis admissions; 1C) shows RSV bronchiolitis hospital admissions; 1D) shows non-confirmed bronchiolitis hospital admissions; 1E) shows preterm bronchiolitis hospital admissions; 1F) shows term bronchiolitis hospital admissions. All bronchiolitis refers to all episodes with a clinical diagnosis of bronchiolitis, irrespective of aetiology. RSV bronchiolitis refers to episodes with an RSV-specific code, assumed to reflect laboratory confirmation in routine NHS testing. Non-confirmed bronchiolitis refers to bronchiolitis without a specific RSV diagnostic code, in which RSV was not detected, or RSV testing was not conducted. The orange line represents observed weekly admissions. The blue line shows the predicted trend based on pre-vaccine data, demonstrating expected admissions in the absence of RSV vaccination. The black dashed line represents the start of maternal eligibility for RSV vaccines. The grey shaded areas represent the COVID-19 exclusion period.

Table S1 Output parameters, primary and sensitivity model for total bronchiolitis admissions in infants under 3 months of age before and after the introduction of the national RSV vaccination campaign in England; primary model uses a quasi-Poisson linear seasonal model; secondary model uses a quadratic-adjusted quasi-Poisson regression analysis.

Model terms*

Model	Term	Estimate	Std error	CI low	CI high	P value
Linear seasonal	dispersion	8.025				
	(Intercept)	-7.937	0.047	-8.029	-7.844	
	interventionborn_pre_vaccination	0.499	0.027	0.447	0.552	<0.001
	interventionborn_within_first_8weeks	0.431	0.046	0.340	0.522	<0.001
	agegrp3-5m	-0.471	0.069	-0.605	-0.336	<0.001
	agegrp6-11m	-0.949	0.068	-1.083	-0.815	<0.001
	year_n	0.057	0.002	0.054	0.061	<0.001
	interventionborn_pre_vaccination:agegrp3-5m	0.057	0.042	-0.025	0.139	0.174
	interventionborn_within_first_8weeks:agegrp3-5m	-0.204	0.090	-0.382	-0.027	0.024
	interventionborn_pre_vaccination:agegrp6-11m	-0.286	0.038	-0.361	-0.211	<0.001
	interventionborn_within_first_8weeks:agegrp6-11m	-0.292	0.097	-0.483	-0.101	0.003
	season_n	-0.050	0.003	-0.056	-0.045	<0.001
Quadratic seasonal	dispersion	7.807				
	(Intercept)	-7.941	0.047	-8.033	-7.850	
	interventionborn_pre_vaccination	0.423	0.029	0.365	0.480	<0.001
	interventionborn_within_first_8weeks	0.396	0.046	0.306	0.487	<0.001
	agegrp3-5m	-0.463	0.068	-0.596	-0.330	<0.001
	agegrp6-11m	-0.936	0.067	-1.068	-0.804	<0.001
	year_n	0.104	0.008	0.088	0.120	<0.001
	I(year_n^2)	-0.004	0.001	-0.005	-0.003	<0.001
	interventionborn_pre_vaccination:agegrp3-5m	0.053	0.041	-0.029	0.134	0.204
	interventionborn_within_first_8weeks:agegrp3-5m	-0.190	0.089	-0.364	-0.015	0.033
	interventionborn_pre_vaccination:agegrp6-11m	-0.292	0.038	-0.366	-0.217	<0.001
	interventionborn_within_first_8weeks:agegrp6-11m	-0.280	0.096	-0.468	-0.092	0.004

season_n:RSVseason	-0.053	0.003	-0.058	-0.047	<0.001

Footnote:
Linear seasonal model: n_admissions ~ intervention * agegrp + year_n + season_n + week * agegrp + offset(offset). Quadratic seasonal model: n_admissions ~ intervention * agegrp + year_n + I(year_n^2) + season_n:RSVseason + week * agegrp + offset(offset). Reference categories: intervention = born post-vaccination; agegrp = <3 months; week = week 1. The week × age group interaction terms are omitted for brevity; full model output is available on request.
n_admissions = count of bronchiolitis admissions, intervention = period of RSV vaccination availability at birth (3-level factor: none, within first 8 weeks of programme, beyond first 8 weeks of programme), agegrp = age group (3-level factor 0-2 months, 3-5 months and 6-11 months), year_n = n^{th} year (continuous: 1(2015),2(2016),...,12(2026)), season_n = n^{th} RSV season (continuous: 0 if outside RSV season weeks 4 to 45, 1(2014/15 season),2(2015/16 season),...,12(2025/26 season) if within RSV season weeks 46 to 3), week = n^{th} week (factor with 52 levels), offset = ln(total live births in England for each specific week, age group and intervention category). Note that in the weeks post-vaccine introduction data for each week were split with some proportion of infants belonging to each intervention category, i.e. the model allowed for the stepped vaccine introduction and the offset accounted for the size of the infant population with/without vaccine-eligible mothers.

Table S2: Estimated episodes of bronchiolitis averted following maternal RSV vaccination, by age group 1 Sep 24- 31 March 2026

Outcome	Under 3 months: Full follow up	Under 3 months: 2024 to 2025 bronchiolitis season	Under 3 months: 2025 to 2026 bronchiolitis season	Under 6 months: Full follow up	Under 6 months: 2024 to 2025 bronchiolitis season	Under 6 months: 2025 to 2026 bronchiolitis season
All bronchiolitis hospital admissions	9,856	2,631	6,549	16,255	3,626	10,923
Critical care bronchiolitis admissions	644	130	443	761	146	512
RSV bronchiolitis hospital admissions	7,088	2,014	4,756	8,963	2,107	6,380
Non confirmed bronchiolitis admissions	5,366	1,645	3,450	10,070	2,500	6,523
Preterm bronchiolitis hospital admissions	1,359	394	844	2,367	539	1,549
Term bronchiolitis hospital admissions	8,518	2,245	5,719	13,908	3,094	9,391

Footnote:

Estimated episodes averted are presented for three follow up periods: full follow up, bronchiolitis season 2024-25, bronchiolitis season 2025 to 2026. Bronchiolitis season estimates include the full October to March period rather than being restricted to model defined peak period. Episodes averted were estimated using difference between observed admissions and predicted admissions under a no-vaccination counterfactual scenario using the primary linear seasonal model. Estimates are independent and derive from separate models and should not be summed

to construct totals. The estimates include episodes averted in infants aged under 3 months and under 6 months of age, as indicated.

Table S3 Alternative model specifications and model comparisons for bronchiolitis hospital admissions

Model	Specification	IRR	95% CI	RMSE	MAE	Comment
Linear seasonal (primary)	Intervention × age + year + season × RSV season + week × age	0.607	0.576–0.640	49.4	31.96	Selected as the primary model based on parsimony, visual assessment of the counterfactual trajectory. Model fit was similar to the quadratic seasonal model.
Quadratic seasonal (sensitivity)	Intervention × age + year + year² + season × RSV season + week × age	0.655	0.619–0.694	48.78	31.73	Marginally lower RMSE and MAE, but requires a more complex quadratic long-term trend and produced a less plausible counterfactual trajectory on visual assessment into the 2025/26 season. Retained this model as a sensitivity analysis to assess robustness.
COVID	Intervention × age + year × post COVID + week × age	0.588	0.548–0.631	56.07	36.37	Allowing the time trend to change following COVID did not improve predictive performance relative to the primary model; balances data too heavily in favour of post- pandemic datapoints
Base	Intervention × age + year + week × age	0.594	0.560–0.630	57.47	36.92	Poorer predictive performance, does not account for seasonality.
Quadratic non-seasonal	Intervention × age + year + year² + week × age	0.622	0.583–0.664	57.43	36.94	Poorer performance and fit; adding a quadratic time trend without accounting for seasonality did not improve performance
No year	Intervention × age + week × age	0.741	0.696–0.789	58.98	40.59	Omitting year produced poorest predictive performance; supports use of year and adjusting for calendar time.

Footnote:

Candidate quasi-Poisson models considered and reviewed during model selection. All models included intervention, age group, week by age group, and an offset based on national birth

registration statistics to approximate population denominators. The linear seasonal model was selected as the primary model and the quadratic seasonal model was selected for the sensitivity analysis. IRR: incident rate ratio; CI: confidence interval; RMSE: root mean squared error; MAE: mean absolute error.
